# “Mouse Clone Model” for evaluating the immunogenicity and tumorigenicity of pluripotent stem cells

**DOI:** 10.1186/s13287-015-0262-3

**Published:** 2015-12-18

**Authors:** Gang Zhang, Yi Zhang

**Affiliations:** Department of Cell & Systems Biology, University of Toronto, 25 Harbord Street, Toronto, Ontario M5S 3G5 Canada; Department of Medicine, Tanz Centre for Research in Neurodegenerative Diseases, University of Toronto, Krembil Discovery Tower, 60 Leonard Avenue, 4th Floor - 4KD481, Toronto, Ontario M5T 2S8 Canada; Division of Nephrology, Massachusetts General Hospital, Harvard Medical School, Harvard University, 149 13th Street, Charlestown, MA 02129 USA; Program in Life Science, New College, University of Toronto, 40 Willcocks Street, Toronto, Ontario M5S 1C6 Canada

## Abstract

To investigate the immune-rejection and tumor-formation potentials of induced pluripotent stem cells and other stem cells, we devised a model—designated the “Mouse Clone Model”—which combined the theory of somatic animal cloning, tetraploid complementation, and induced pluripotent stem cells to demonstrate the applicability of stem cells for transplantation therapy.

In 2011, Zhao et al. [1] reported that, compared with embryonic stem cells (ESCs), induced pluripotent stem cells (iPSCs) showed more immune rejection and overexpression of immunogenicity-related genes. This report shed shadows on the great promise of iPSCs as the renewable sources of autologous cells for regenerative medicine. Because of the different origins and treatment of ESCs and iPSCs [2–4], it is reasonable that there are some differences between them, even though iPSCs can eventually give rise to viable mice by tetraploid complementation [5, 6] and iPSC mice can make further mice [7]. These data demonstrate that iPSCs could have the same pluripotency as ESCs. At present, iPSCs can be generated with several different protocols, including retroviral infection [3], lentiviral transduction [8], nonviral minicircle vector transfection [9], and so forth. It is true that a heterogeneic situation will always be found in iPSCs. Polo et al. [10] reported that iPSCs derived from different cell types, such as mouse fibroblasts, hematopoietic cells, and myogenic cells, exhibited distinct transcriptional and epigenetic patterns. Furthermore, the cellular origin influences the in vitro differentiation potentials of iPSCs. But continuous passaging of iPSCs largely attenuates these differences. These data indicate that the heterogeneity of iPSCs might be decreased by further reprogramming with more passaging [10]. Great achievements have so far been made in the application of iPSC transplantation. For example, the successful corrections of sickle cell anemia, Fanconi anemia, and tyrosinemia [11–13] via the transplantation of iPSC-derived differentiated cell types into diseased mouse models.

The shortcoming of this research is to use the same strain of C57BL/6 (B6) mice as recipients to test the immune rejection of the iPSCs, derived from mice which are within the same strain but are not the same individual mice, between the donors of iPSCs and the recipients [1]. For example, C57BL/6 mice are an inbred strain and are nearly identical to each other in genotype due to long inbreeding. Although transplantations between inbred mice have been conventionally used as a model to test immune acceptance and are considered autologous transplantation, and in some sense they are in theory equivalent to autologous human tissue/cell transplantations, this is not completely true. Here, it is worthy of note that inbred mice are nearly identical in genotype, but they are not exactly the same. Furthermore, even though it is well known that inbred mice can fully accept the same inbred strain mouse organs, including skin grafts, and therefore are a rigorous model to assess immune tolerance, this might not be the same in the case of stem cell transplantation therapy, such as iPSC and ESC transplantations. It is well known that immune rejection exists not only species specifically, but also individual specifically, including within the same strain, due to alloimmunity [14].

To evaluate the applicability of iPSCs for autologous transplantation, we devised a novel animal model by combining the theory of animal cloning [15], the protocol of tetraploid complementation [16], and the induction of iPSCs [3, 4, 7] to establish a large number of cloned mice derived from a single inner cell mass (ICM) of mouse blastocyst (Fig. 1). The reasons for using ESCs as the first step include, first of all, that we can compare the similarity and difference between ESCs and iPSCs of the same origin, because they are genetically from the same mouse blastocyst. In addition, by using ESCs as the starting point, we can produce both ESC mice and iPSC mice, so we can compare them to determine whether they are exactly the same or have some differences. Theoretically, these cloned mice are exactly identical to each other. Therefore, truly autologous stem cell transplantations can be performed between them. Moreover, because the starting point of the cloned mice is the ESCs, the transplantation characteristics among ESCs, iPSCs, and tissue-specific stem cells can be analyzed with this model. Adopting this clone of mice as a unique source, iPSC lines can be induced and established. At the same time, other stem cells of different tissues can also be isolated. As a result, the iPSCs and tissue-specific stem cells, together with their progenies of different differentiated stages, can be tested by transplanting them into the mice of the same clone to achieve truly autologous transplantation to mimic human patient-specific iPSCs for the patients (Fig. 1). In addition, during the reprogramming of iPSCs, some genetically different cell lines with different pluripotency can be generated by different protocols and other unknown reasons [1, 3, 5–7]; therefore, this model can also help to answer which lines are better for therapeutic applications with less immune rejection. Furthermore, the availability of plenty of the same-origin clone of mice can further allow the investigators to examine the therapeutic advantages of various kinds of tissue-specific stem cells with different differentiated stages. The discovery that mice can be serially recloned for up to 25 generations with consistent cloning efficiency and without evident genomic errors indicated that mice possibly can be recloned limitlessly by the somatic nuclear transfer method [17]. Thus, in theory, mice can also possibly be limitlessly recloned by tetraploid complementation strategy with iPSCs. Hence, iPSCs can be isolated and induced limitlessly from these iPSC mice.

**Fig. 1:**
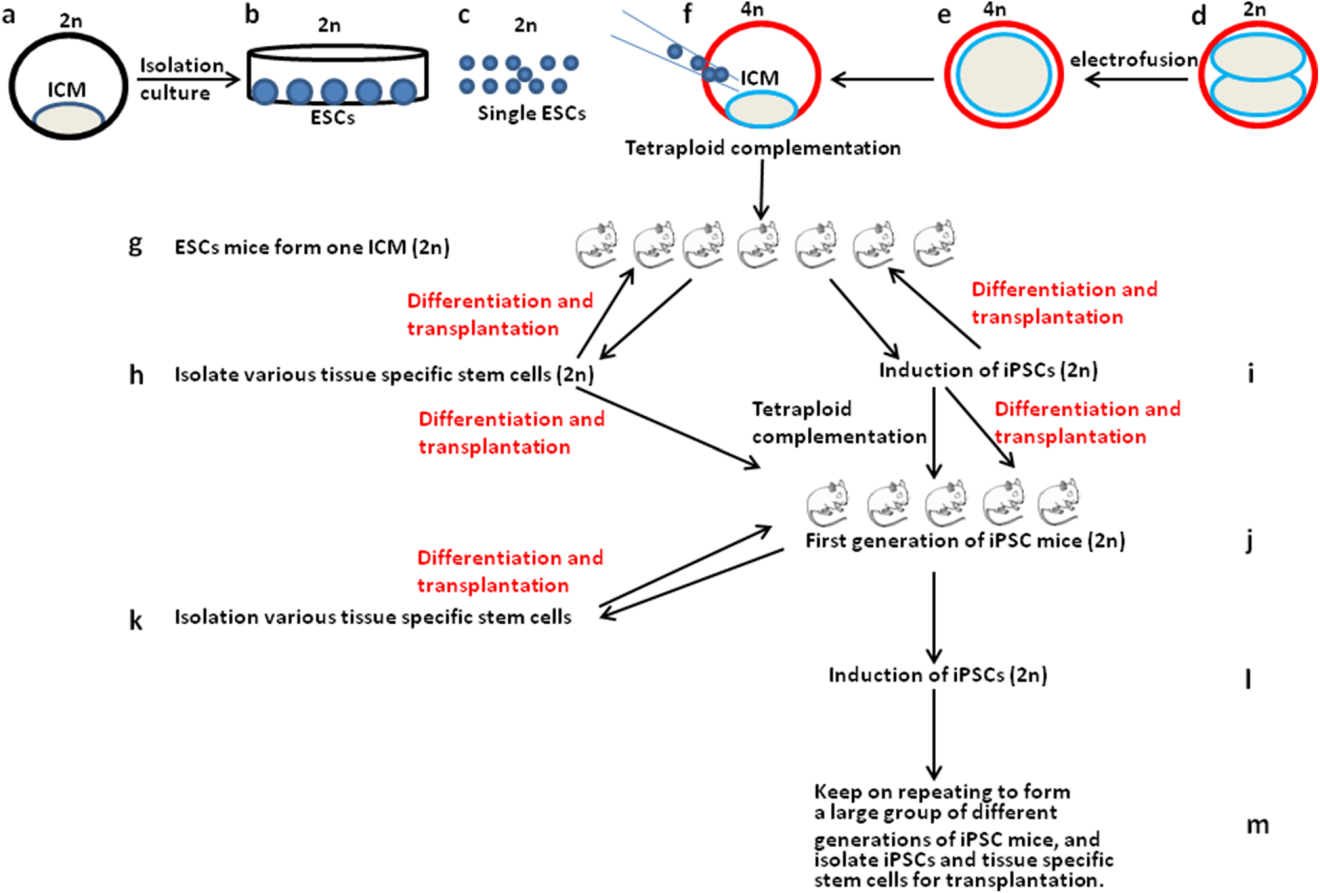
Scheme of the “Mouse Clone Model” for stem cell transplantation. **a** One unique 2n mouse blastocyst. **b** *2*n ESCs isolated and cultured from **a**. **c** *2*n ESCs separated from **b** for microinjection. **d** Many 2n mouse two-cell embryos from the same or different mouse strains with **a**. **e** Many 4n one-cell embryos fused from **d**. **f** Many 4n blastocysts generated from **e** and injected with 2n ESCs from **a** to produce ESC mice by tetraploid complementation. **g** Mouse clone from 2n ESCs, theoretically all the same as each other. **h** Various tissue-specific stem cells isolated from **g**, using these stem cells to transplant **g** mice; theoretically transplanted into “themselves”. **i** Many induced iPSCs from **g** with different protocols [3, 4, 7]; theoretically, they should be the same as **g**, but for epigenetic reasons there are some differences between them. These iPSCs can be differentiated or directly transplanted into the mice of **g**. This step could identify “good” iPSC lines from “bad” iPSC lines based on the data of immune rejection and tumor formation, using the “good” iPSC lines which do not form tumor and do not have, or have less, immune rejection to generate iPSC mice. **j** First generation of iPSC mice generated from different iPSC lines from **i** by tetraploid complementation. This step can identify the “good” iPSC lines which can generate live mice from those which cannot. Combined with the data from the transplantation **i**, we can identify “good” iPSC lines, which can generate live mice, cannot form tumor, and do not have, or have less, immune rejection. **k** Various tissue-specific stem cells isolated from first-generation iPSC mice. These iPSC mice-derived tissue-specific stem cells can be differentiated or directly transplanted into the ESC-derived mice and iPSC-derived mice to investigate their efficacies according to the commonly agreed criteria. **l** Induced iPSC lines from first-generation iPSC mice. These lines are again investigated by transplanting into ESC mice, first-generation iPSC mice, and tetraploid complementation to produce the second generation of iPSC mice. **m** Keep on repeating to form a large group of different generations of iPSC mice, and isolate iPSCs and tissue-specific stem cells for transplantation. Using this model and strategy, a large clone of mice will be established from a unique 2n mouse blastocyst, for the investigation of stem cell therapy. *ESC* Embryonic stem cell, *ICM* Inner cell mass, *iPSC* Induced pluripotent stem cell

Although autoimmune diseases exist, and in some cases the transplantation tolerance observed in mouse models cannot be validated in large animal models and human due to different species, this strategy will at least theoretically provide a clone of truly identical mice for stem cell therapy investigation. Using this method, a clone of immortal mice can be established eventually, and they will benefit the stem cell therapy research greatly in a mouse–mouse pattern. In conclusion, this model—the “Mouse Clone Model”—can provide an unprecedented strategy to vigorously assess the safety of mouse ESCs, iPSCs, and various kinds of tissue-specific stem cells in “themselves”. Using this model in mouse stem cell transplantation, we can, in theory, avoid using immunodeficient mice and inbred mice to study stem cell transplantation because of their disadvantages (Table 1). For human stem cell therapy, because we cannot directly test iPSCs and other stem cells in the human body, some of the major concerns such as immune rejection and tumor formation after transplantation cannot be determined vigorously. With this model, we can at least investigate whether or not the in vitro culture procedure, induction, and differentiation might cause problems for immune rejection and tumor formation in mice. If we can prove experimentally that the in vitro culture, induction, and differentiation procedure cannot induce immune rejection and cancer formation using this mouse model, then we can gain more confidence that the patient-derived iPSCs might be used for transplantation. Certainly, there are many differences for stem cell transplantation between mice and human, but it is worth trying. This model will therefore lay important foundations for personalized patient-specific tissue stem cells and iPSCs for transplant therapy.

**Table 1:** Advantages and disadvantages of different mouse models

Mouse models	Features	Advantages	Disadvantages
Inbred mouse strain model	Nearly identical to each other genetically	Many different strains are available, such as C57BL/6, etc.	Not exactly the same with each other genetically and have the potential to induce immune reaction
Immunodeficient mouse model [29]	Deficient immune system for various reasons	Many different strains are available, such as nude-mice strains, etc.	Some transplanted stem cells might result in tumors due to the weakened immune system
“Mouse Clone Model”	Theoretically identical with each other, and exactly the same between transplanted stem cells and mouse recipients	In theory, no immune rejection reaction, and cannot form tumor due to the normal immune system	Need tedious work and high techniques to generate a clone of mice

Another concern about stem cell transplantation therapy is tumor formation. The discovery of cancer stem cells (CSCs) demonstrated that, in the hierarchy of the cancer cell population, there is a subset of stem-like tumor cells that have the ability to self-renew and differentiate into the diverse tumor cells [18–21]. There are good reasons to believe that during the in vitro culture of stem cells, and particularly the in vitro induction with viral integrations of iPSCs, stem cells are exposed to risks for some mutations and epigenetic changes [22–24]. Thus, after transplantation into recipients, the stem cells, in some cases, can result in various tumors [25–28]. Different immune-deficient mice are commonly used as recipients, to facilitate the integration of transplanted stem cells. On the other hand, due to their deficiency of immunity, some stem cells can accumulate to form tumors when they undergo clonal evolution [25, 26]. Using the “Mouse Clone Model” for mouse stem cell transplantation, theoretically—because the recipients and stem cells and their derivatives are “selves” and have “normal” immunity—the risks of forming tumors would be decreased radically. This “Mouse Clone Model” could therefore not only reduce the immune rejection radically, but also decrease the risks of tumor formation significantly. The advantages and disadvantages of different mouse models are briefly presented in Table 1; for more details, refer to [29].

Ideally, the applicable iPSCs for transplantation therapy should have full pluripotency, such as supporting full iPSC mice. This is the gold standard for full pluripotency for mouse ESCs and iPSCs. In addition, the iPSCs and their differentiated stem cells should not form tumors. Compared with currently used animal models for intraspecies stem cell transplantation, the “Mouse Clone Model” is much more advantageous for the following investigations: to investigate the immune rejection of established stem cell lines, including iPSCs and various tissue-specific stem cells, and to select suitable stem cell lines and cloned mice based on the immune rejection data; to investigate the tumor formation of iPSCs and other stem cell lines and to select suitable stem cells and cloned mice that possess low or absent tumorigenicity based on the tumor formation assay [27]; to investigate, after the selection of applicable stem cells and mice, the appropriate stages of different stem cells for their transplantation; and to genetically decipher the mechanisms of immune rejection and tumor formation more precisely. Because the stem cells and the mouse clone are biologically “selves”, this model will provide much stronger and direct evidence for stem cell therapy, and further give instructions for patient-specific iPSC-based therapy clinically. We suggest that stem cell researchers all over the world should investigate the applicability of iPSCs and tissue-specific stem cells for therapeutic applications taking advantage of this model.

## Abbreviations

CSC: Cancer stem cell
ESC: Embryonic stem cell
ICM: Inner cell mass
iPSC: Induced pluripotent stem cell
